# Stigma towards People with Mental Illness among Portuguese Nursing Students

**DOI:** 10.3390/jpm12030326

**Published:** 2022-02-22

**Authors:** Júlio Belo Fernandes, Carlos Família, Cidália Castro, Aida Simões

**Affiliations:** 1 Escola Superior de Saúde Egas Moniz, Caparica, 2829-511 Almada, Portugal; carlosfamilia@egasmoniz.edu.pt (C.F.); ccastro@egasmoniz.edu.pt (C.C.); aidasimoes@gmail.com (A.S.); 2 Grupo de Patologia Médica, Nutrição e Exercício Clínico (PaMNEC)—Centro de Investigação Interdisciplinar Egas Moniz (CiiEM), 2829-511 Almada, Portugal; 3 Centro de Investigação Interdisciplinar Egas Moniz (CiiEM), 2829-511 Almada, Portugal; 4 Molecular Pathology and Forensic Biochemistry Laboratory (MPFBL), Centro de Investigação Interdisciplinar Egas Moniz (CiiEM), Monte de Caparica, 2829-511 Caparica, Portugal

**Keywords:** mental disorders, mental illness, social stigma, nursing, students

## Abstract

Stigma is a substantial obstacle when caring for people with mental illness. Nursing students’ negative attitudes towards people with mental illness may impact the quality of care delivered and consequentially patient outcomes. In this study, we assessed the stigmatising attitudes and beliefs of nursing students towards people with mental illness and examined its relationship with several psycho-socio-demographic variables. This was a quantitative, cross-sectional descriptive correlational study, which was developed with a non-probabilistic convenience sample of 110 nursing students. Stigmatising attitudes and beliefs were assessed using the Portuguese version of the Attribution Questionnaire AQ-27. Results show that the dimensions of stigma with higher scores were help, pity, coercion and avoidance. However, significant differences were only observed depending on the year of study (fourth-year students, who already had clinical placements in this area, are less likely to show stigma), the relationship (family is less prone to show coercion), the history of mental health treatment (students with a history of mental health treatment have more tendency to help) and whether they considered working in the mental health field (students who have considered working in this field are less prone to show anger, avoidance and think of patients as dangerous). Therefore, we conclude that education in a classroom setting alone is not enough to reduce stigma in nursing students, clinical placement in the area is required to achieve such results. It is thus essential to improve nursing curricula worldwide so that students are exposed to both psychiatric nursing theory and clinical practice in the first years of the nursing degree.

## 1. Introduction

Mental illness emerges as a consequence of mental disorders and is usually manifested by abnormal thoughts, emotions, perceptions, behaviours and relationships with others. Mental illness is a highly heterogeneous group of disorders, which include depression, psychosis, dementia and developmental disorders, among others [1]. Mental illness is presently recognised as a severe health problem that affects the life of one in every three Europeans [2] and is one of the leading causes of disease-related burden worldwide [3].

The knowledge and attitude of the general public towards people with mental illness differs depending on the type of illness. For example, several studies described a higher propensity for people to maintain a greater social distance from a person with schizophrenia when compared with someone with an anxiety disorder or depression [4,5,6]. This tendency was also verified in a study that compared physicians’ attitudes towards people with schizophrenia and depression [7].

When a stigma towards a specific population is identified, it is vital to develop interventions to mitigate distress and shift harmful behaviours that may compromise this population’s wellbeing [8]. To interrupt the stigmatisation process, it is essential to assess the presence of stigma and promote educational anti-stigma campaigns, presenting factual information about the stigmatised condition, and to develop contact-based strategies aimed to facilitate the interaction and connection between groups [9,10,11].

According to the Health Stigma and Discrimination Framework, stigma spans across the socio-ecological spectrum in the form of stereotyping, prejudice and discrimination [11]. Stigma is, therefore, a multidimensional concept that includes different dimensions (cognitive, affective and behavioural) that operate at the micro-level (singular person), meso-level (social networks), macro-level (cultural or institutional) and that can occur consciously (explicitly) or unconsciously (implicitly) [11,12].

The stigmatisation process can unfold into different domains, including drivers and facilitators, stigma marking and stigma manifestations, all of which have a direct effect on several outcomes among populations targeted by stigma [11]. The first domain includes factors that drive or facilitate the stigmatisation process and are usually conceptualised as inherently negative and might diverge according to the targeted population’s health condition [11]. Stigma marking and manifestations are usually displayed as a series of stigma experiences (i.e., experienced stigma and discrimination) and practices (i.e., stereotypes, prejudice and discriminatory attitudes) [11]. In addition, these experiences can also induce self-stigma, which occurs when stigmatised group members internalise the negative societal beliefs and feelings and suffer numerous negative consequences as a result [11,12,13].

Stigmatisation experiences and the inherent associated labelling, usually result from the lack of knowledge about mental illnesses and contact with cases of discrimination and negative attitudes from society [6]. These negative attitudes towards people with mental illness, can lead to social isolation and delay or prevent these persons from getting help and treatment or even interacting with essential community services [7,8]. In fact, recently, the Health Stigma and Discrimination Framework postulated that stigma manifestations will have an impact on several outcomes for affected populations. These might include access to essential services like justice and healthcare services and influence outcomes for organisations, including the availability and quality of health services [11].

Healthcare professionals, contrary to expectations, are also prone to display negative and stigmatising attitudes and behaviours towards people with mental illness [9], which leads to mismanagement and low attention to patients, undoubtedly affecting their interaction, and ultimately leading to a lack of support, acceptance and of an appropriate and adequate care of these patients [7,8,14,15]. Positive attitudes toward people with mental illness are thus a prerequisite healthcare professionals must demonstrate in order to provide quality care to these patients. For example, having adequate expectations about the behaviours and traits of the disease and being able to correctly identify and avoid incorrect messages and societal misconceptions [14,16].

Nursing students share the same misconceptions towards people with mental illness as the general public, and those with a high level of stigma often display discomfort, anxiety and fear when caring for people with mental illness [17,18]. In fact, a recent study identified stigmatised misconceptions about people with mental illness in nursing students, such as the assumption of them being dangerous and having worse prognoses [19]. Other studies also identify that nursing students believed that people with mental illness needed to be segregated from the community [20,21] and had more difficulty expressing compassion for those patients [20].

Current evidence indicates that theoretical preparation and clinical placements in mental health units, effectively reduce the stigma of nursing students towards people with mental illness [22,23]. Furthermore, it can represent an opportunity to attract students into this field [22]. In fact, presently, there is a severe shortage of qualified professionals, trained to provide timely and effective treatment for mental health patients, roughly less than 9 workers per 100,000 population [24]. For these reasons, it is crucial to deconstruct nursing students’ misconceptions regarding people with mental illness as their attitudes and beliefs will certainly have a major impact on the career path they will take after graduation [17,25]. Educational institutions are thus in a privileged position to address this problem by elucidating these misconceptions and promoting the idea that people with mental illness are not entirely responsible for their condition and cannot control it [17]. 

Considering that the first action to intervene in order to mitigate the stigmatisation process is the assessment of the situation, with this study, we aim to assess the stigma nursing students have towards people with mental illness and examine the relationship between stigma and psycho-socio-demographic variables, so that intervention actions that allow changing this panorama can be developed.

## 2. Methods

### 2.1. Design

This is a quantitative study that was conducted using a cross-sectional descriptive correlational web-based survey design.

### 2.2. Study Setting

The study took place in a Portuguese private Higher School of Health in the region of Lisbon and Tagus Valley. A non-probabilistic convenience sample was used where all undergraduate students attending the nursing degree course were invited to participate in the study through their school e-mail address.

### 2.3. Data Collection

The data collection instrument was written in Portuguese and included the study information page, psycho-socio-demographic questionnaire and Attribution Questionnaire (AQ-27) [26]. All questions were transcribed into Google Forms™ and were applied from May to July 2020.

The psycho-socio-demographic questionnaire collected background information such as age, sex, religion, place of residence, year of study, prior contact with mental illness, history of mental health treatment and if the student ever considered working in the field of mental health.

The AQ-27 [26] questionnaire was used to assess stigma towards people with mental illness. For this study, we used the Portuguese version of the AQ-27, which was previously translated and validated for the Portuguese population by Sousa, Queirós, Marques, Rocha and Fernandes [27], having previous studies performed on the Portuguese population with this questionnaire yielded Cronbach alpha values of reliability of 0.88 [28], 0.76 [29] and 0.83 [30]. The AQ-27 is a self-administered questionnaire that consists of a brief vignette about a hypothetical person with schizophrenia, chosen among the provided vignettes, specifically not to influence emotional reactions from the participants. This vignette was followed by a set of 27 questions addressing one of nine subscales or dimensions of stigmatising attitudes and beliefs towards people with mental illness (Anger, Avoidance, Blame, Coercion, Dangerousness, Fear, Help, Pity and Segregation). The score for each subscale is obtained by the sum of the three questions corresponding to that subscale. Each question is answered on a Likert-type scale that ranges from 1 to 9. The AQ-27 has no defined threshold score for levels of stigma, for this reason, the score obtained in each dimension of stigma should be interpreted comparatively [26].

### 2.4. Data Analysis

The statistical analysis of the questionnaires was performed using the R language and environment for statistical computing v. 4.1.2 [31], with RStudio v.2021.09.0 [32] as the integrated development environment. Only surveys with all questions answered were analysed.

Descriptive statistic measures of count, mean, standard deviation, median, minimum, maximum and range were computed for sample characterisation, using the function table1 from the table1 v.1.4.2 library [33] for R. Values of minimum, maximum, mean and standard deviation were also computed for each question, and each dimension of stigma existing in the questionnaire, which was summarised in a table using the kable function from knitr v.1.36 library [34,35,36] for R.

Linear models were developed for each dimension of stigma with the categorical variables of Year of study, Sex, Religion, Residence, Relationship (know someone with mental illness and their relationship to that person), History of mental health treatment and Considered working in the mental health field as predictors, using the lm function provided by the R base library. The Year of study included four levels (first, second, third and fourth years), as well as Relationship (None, Acquaintance, Friend or Family), Sex consisted of two levels (Male or Female), as well as Religion (Yes or No), Residence (Rural or Urban), History of mental health treatment (Yes or No) and Considered working in the mental health field (Yes or No).

Model assumptions were verified through the visual observation of the residuals plot, Q-Q plot, Index plot and Histogram provided by the resid_panel function of the ggResidpanel v.0.3.0 library [37] for R. In addition, assumptions of normality and homoscedasticity of the standardised residuals were also formally evaluated with the Shapiro–Wilk and the Breush–Pagan tests, respectively. The former is provided by the shapiro.test function of the R stats v. 4.1.2 library, and the latter was provided by the bptest function of the lmtest v. 0.9.38 library [38] for R. Whenever these assumptions were violated, the dimension of stigma in question was transformed with the Box-Cox transformation, with a lambda value determined computationally by the powerTransform function of the car v.3.0.11 library [39] for R, after which a new linear model was developed and assumptions reverified as previously described.

Subsequently, a factorial analysis of variance (ANOVA) of type 2 was performed using the Anova function from the car library for R, and the results were summarised in a table using the apa.aov.table function from the apaTables v.2.0.8 library [40] for R. Multiple comparisons with Tuckey contrasts were performed for each main effect identified to have at least two groups with significant differences, using the glht function of the multcomp v.1.4.17 library [41] for R. For all statistical tests a level of significance of 0.05 was considered.

### 2.5. Ethical Considerations

Ethics approval was obtained from the Board of Directors and the Institutional Ethical Review Committee of the Education Institution involved (Date: April 2020 ID: 884). The survey’s first page contained a clarification of the objectives and procedures of the study and the guarantee that confidentiality and anonymity of the data were assured by the researchers. Participants would need to accept and agree to the online informed consent in order to complete the survey. The survey was set up so that participants had to answer “Yes” or “No” indicating that they had read the consent information and agreed to participate. Only the participants who answered “Yes” to the informed consent question were directed to the research survey. Participants who answered “No” to the informed consent question were directed to the end of the survey. Participants were free to decide not to answer any question, change or review their responses, or voluntarily quit at any time. To comply with the ethical principles of anonymity and confidentiality, all data collected were free of any personally identifying information, including any form of electronic identifiers.

## 3. Results

A total of 110 nursing students have participated in this study, obtaining a response rate of 51.2%. Most participants were female (91.8%), with a mean age was 22 years (SD = 4.47). From these, 90.9% lived in a predominantly urban environment, 50.9% knew or had direct contact with people with mental illness, 33.6% had a history of mental health treatment and 52.7% had already considered the possibility of working in the mental health field after graduating (Table 1).

Table 2, presents the mean and standard deviation obtained for each dimension of stigma, divided by each level of the various psycho-socio-demographics under study, as well as the overall measures for these dimensions. In addition, values of minimum, maximum, mean and standard deviation, obtained for each of the AQ-27 items are presented in Table A1. 

A factorial ANOVA of type 2 was performed to compare the main effects of Year of study, Sex, Religion, Residence, Relationship, History of mental health treatment and Considered working in the mental health field on the score of each subscale or dimension of the stigmatising attitudes and beliefs towards mental illness defined in AQ-27 (Anger, Avoidance, Blame, Coercion, Dangerousness, Fear, Help, Pity and Segregation), detailed ANOVA tables can be found in the Appendix A (Table A2, Table A3, Table A4, Table A5, Table A6, Table A7, Table A8, Table A9 and Table A10).

The main effect for Year of study showed to significantly affect all dimensions, Anger, Avoidance, Blame, Coercion, Dangerousness, Fear, Help, Pity and Segregation (*F*(3.98) = 6.27, *p* = 0.001, partial η^2^ = 0.16); *F*(3.98) = 7.97, *p* ≤ 0.001, partial η^2^ = 0.20; *F*(3.98) = 22.29, *p* ≤ 0.001, partial η^2^ = 0.41; *F*(3.98) = 3.18, *p* = 0.027, partial η^2^ = 0.09; *F*(3.98) = 7.04, *p* ≤ 0.001, partial η^2^ = 0.18; *F*(3.98) = 7.94, *p* ≤ 0.001, partial η^2^ = 0.20; *F*(3.98) = 6.22, *p* ≤ 0.001, partial η^2^ = 0.16; *F*(3.98) = 8.01, *p* ≤ 0.001, partial η^2^ = 0.20; and *F*(3.98) = 8.87, *p* ≤ 0.001, partial η^2^ = 0.21, respectively). Subsequent multiple comparisons showed significant differences between the 1st and 2nd Year for Blame (t = 3.18, *p* = 0.010). Borderline significant differences between the 1st and the 3rd Year for Anger (t = −2.40, *p* = 0.084) were shown. Significant differences between the 1st and 4th year for Anger, Avoidance, Blame, Dangerousness, Fear, Help, Pity and Segregation (t = −3.91, *p* = 0.001; t = −4.05, *p* ≤ 0.001; t = −5.56, *p* ≤ 0.001; t = −3.70, *p* = 0.002; t = −3.31, *p* = 0.007; t = 3.89, *p* ≤ 0.001; t = −3.34, *p* = 0.006; and t = −4.32, *p* ≤ 0.001, respectively) were shown. Borderline differences between the 2nd and 3rd Year for Fear (t = −2.59, *p* = 0.053) were shown. Significant differences between the 2nd and 4th year for Anger, Avoidance, Blame, Coercion, Dangerousness, Fear, Help, Pity and Segregation (t = −3.34, *p* = 0.007; t = −4.56, *p* ≤ 0.001; t = −7.80, *p* ≤ 0.001; t = −3.08, *p* = 0.014; t = −4.31, *p* ≤ 0.001; t = −4.83, *p* ≤ 0.001; t = 3.78, *p* = 0.002; t = −4.84, *p* ≤ 0.001; and t = −4.78, *p* ≤ 0.001, respectively) were shown. There were also significant differences between the 3rd and 4th year for Avoidance, Blame, Dangerousness, Fear, Help, Pity and Segregation (t = −2.80, *p* = 0.031; t = −6.45, *p* ≤ 0.001; t = −3.43, *p* = 0.005; t = −2.63, *p* = 0.048; t = 2.59, *p* = 0.053; t = −3.28, *p* = 0.008; and t = −3.73, *p* = 0.002, respectively).

The main effect for Religion was shown to significantly affect the dimension Help (*F*(3.98) = 6.01, *p* = 0.016, partial η^2^ = 0.06) and to borderline significantly affect Coercion (*F*(3.98) = 3.60, *p* = 0.061), indicating significant or borderline significant differences between students who are religious and those who are not for these dimensions.

The main effect of Relationship was shown to significantly affect the dimension Coercion (*F*(3.98) = 4.73, *p* = 0.004, partial η^2^ = 0.13). Subsequent multiple comparisons showed significant differences between the None and Family and borderline significant differences between Friend and Family (t = −3.37, *p* = 0.006; and t = −2.49, *p* = 0.066, respectively) for this dimension.

The main effect of Considered working in the mental health field was shown to significantly affect the dimensions of Anger, Avoidance and Dangerousness (*F*(1.98) = 14.39, *p*-value = 0.001, partial η^2^ = 0.13; *F*(1.98) = 11.59, *p* = 0.001, partial η^2^ = 0.11; and *F*(1.98) = 5.25, *p* = 0.024, partial η^2^ = 0.05, respectively) and to borderline significantly affect Segregation (*F*(1.98) = 3.01, *p*-value = 0.086, partial η^2^ = 0.03), which indicates significant differences between the group of students who had considered working in mental health and the group of students who did not for these dimensions.

The main effect for History of mental health treatment was shown to significantly affect the dimension Help (*F*(3.98) = 10.18, *p* = 0.002, partial η^2^ = 0.09) and to borderline significantly affect Coercion (*F*(1.98) = 4.73, *p*-value = 0.092, partial η^2^ = 0.03), which indicates significant differences between students who had previously received treatment for any mental illness and those who did not for these dimensions.

## 4. Discussion

The overall results from this study show that some level of stigma towards people with mental illness is present across all dimensions of stigma, which is in line with previous studies that also found explicit stigma in nursing students regarding people with mental illness [19,20,21,22,23].

Nursing students that participated in the present study showed higher scores in the stigmatisation attitudes and behaviours subclasses of Help, Pity and Coercion. This agrees with what was observed in a previous study with Portuguese students from different healthcare courses, which also revealed a high level of Pity, especially among nursing students [21]. These high scores obtained for Help and Pity may imply that students have the tendency to aid and demonstrate kindness towards people with mental illness. However, as reported in previous studies, this result may also point to a paternalistic view of people with mental illness because they portray them as not competent and in the need of help [42,43,44]. In addition, the high scores obtained for Coercion may indicate that the students prioritise the compliance to pharmacological treatments and routine medical appointments on the person’s wellbeing, as they do not see these patients as having the capability of making healthcare-related decisions, and thus students tend to avoid empowering these patients and to ignore their involvement in the process, similarly to what was observed in previous studies [20,21,45]. In particular, this was observed by Querido et al. [21], with Portuguese students from different healthcare courses, who also revealed a high level of Pity, especially among nursing students.

Another dimension of stigmatisation with high scores was Avoidance, which is interesting, considering that high scores were also observed for Help and that low scores were observed for Dangerousness and Segregation. This may imply that despite the students do not fear or perceive people with mental illness as dangerous or necessary to be segregated and though they recognise these patients need help, they still try to avoid them. Interestingly, fourth-year students which were exposed to a second curricular unit of psychiatric nursing theory and the clinical placement and with a formal theory training, show a considerably lower score for avoidance than colleges from different class years. These findings show that contrary to most nursing curricula, the inclusion of a clinical placement prior to the beginning of the 4th year which puts the students in direct contact with these patients, is extremely important to mitigate some of the preconceptions they might have, leading to Avoidance attitudes and behaviours.

However, our respondents, regardless of the year, reported lower scores in the dimensions of Anger, Blame, Segregation, Fear and Dangerousness than in the remaining dimensions, which contrast with the findings of Querido et al. [21], where students showed similar scores across all dimensions of stigma. Exposure to psychiatric nursing theory in their first year of the degree may explain the low scores obtained in these factors, which allows them to perceive people with mental illness differently, since the very beginning of their academic path. These results are consistent with data from current and previous studies that identified that a higher load of theoretical preparation in the nursing curricula was usually associated with fewer stigmatising attitudes towards people with mental illness [22,46].

Significant differences, with considerable effect sizes, were only identified in the stigmatisation attitudes and behaviours between different class years. It is important to emphasise that first-year students were only exposed to a curricular unit of psychiatric nursing theory, and only the fourth-year students were exposed to a second curricular unit of psychiatric nursing theory and a practical clinical placement in the psychiatric-mental health field. As suggested by the American Psychiatric Nurses Association Education Council [47], this theoretical class curriculum has several vital contents for student nurses’ skill development, required to provide quality care for people with mental illness, namely: principles of cognitive, emotional and psychological growth; therapeutic interventions for patients and families experiencing, or at risk for, psychiatric disorders; appropriate affective and cognitive responses to patients; communication with patients experiencing common psychiatric symptoms; and de-escalation of aggressive behaviour. However, at our institution, we decided to complement the students’ formation of this area by introducing a second curricular unit focused on the concepts of psychopathology, neurological basis of psychiatric-mental health practice, pharmacotherapeutics and basic principles of pharmacology, clinical decision-making and health promotion and illness prevention in a second curricular unit. This exposure to a complementary theoretical curricular unit and to the clinical placement practise seems to be the differentiating factor that impacts the overall stigma scores for our students. This is consistent with prior literature that revealed that, in nursing students, practical experience with mental illness patients is related to fewer stigma attitudes and behaviours [22,48]. Interestingly, stigma scores obtained from first-year students are generally lower than the scores obtained from second and third-year students, which indicates that exposure to psychiatric nursing theory alone does not have a long-standing impact on the students’ attitudes towards people with mental illness, and thus by itself is not the key factor to eliminate or reduce stigma in the long term.

In the analysis of the dimension Coercion, even though the present study did not assess kinship, it is essential to emphasise that the score of Coercion was lower among students who have a family member with mental illness.

In the Avoidance dimension, it was identified that students who have considered working in the mental health field show less tendency to avoid people with mental illness. This trend was also identified in the dimensions Anger and Dangerousness. This result seems logical as by choosing to work in the mental health field, students opt for a nursing field where they think they will feel comfortable providing their care and establishing a nurse–patient relationship. Therefore, they are more available to interact with people with mental illness [49].

This study also shows that students who have a history of mental health treatment are less prone to show anger and tend to help people with mental illness, probably because students can empathise easier with these patients. In addition, by experiencing the need to undergo psychiatric or psychological treatment, students become more aware of the difficulties patients are experiencing, so they can show less anger and demonstrate more willingness to help. This is in sharp contrast with a previous study conducted among Indonesian nursing students, which showed that having experienced a mental illness was not correlated with the students’ attitudes toward mental illness [46].

Nonetheless, this study has some limitations that deserve to be mentioned: (i) the sample limited size and composition; (ii) its limitation to only one nursing school, which limits possible generalisations to other contexts and settings [50]; (iii) the use of a self-report survey as opposed to direct behavioural observations, as the participants’ answers may not represent their actual behaviours due to social desirability [51] (however, due to the online nature of the survey and since no identifying data were collected, we believe that this imitation might have been mitigated); and (iv) even though the survey’s first page clarified that the study aimed to assess the stigma of nursing students towards people with mental illness, the AQ-27 contains a brief vignette about a hypothetical person with schizophrenia. Therefore, the findings from this study may need to be interpreted as being attitudes towards people with schizophrenia rather than mental illnesses in general.

## 5. Conclusions

This study assessed stigma towards people with mental illness among nursing students and examined the relationship between nursing students’ stigma and psycho-socio-demographic variables.

Findings revealed that nursing students mainly show stigmatising attitudes in the form of help, pity, coercion and avoidance. Findings also indicate that clinical placement may play a more vital role than acquired theoretical knowledge alone. All students received formal training in their first year of study, but only the students who completed the clinical placement showed decreased stigmatisation attitudes and behaviours. Hence it is clear that combining theoretical education with the practice of clinical placement is one possible and effective approach to help reduce nursing students’ stigma towards these patients. By being exposed to psychiatric nursing theory, students are expected to increase their knowledge about mental illness, treatment and nursing care. Associating this knowledge to the practise of the clinical placement can increase a positive attitude towards people with mental illness. These findings have important implications for academic education, reinforcing the need to develop effective strategies to help advance the fight against stigma towards people with mental illness. The improvement in nursing curricula with the development of psychiatric nursing theory and clinical placement in the first years of study can be an effective strategy, as shown by this work. Nonetheless, further investigations focused on the effect of this strategy are essential for a better comprehension of their impact on the nursing students’ stigmatisation attitudes and behaviours towards people with mental illness.

## Figures and Tables

**Table 1:** Socio-demographic characteristics of the sample.

	1st Year (N = 36)	2nd Year (N = 24)	3rd Year (N = 29)	4th Year (N = 21)	Overall (N = 110)
Sex					
Female	33 (91.7%)	24 (100%)	26 (89.7%)	18 (85.7%)	101 (91.8%)
Male	3 (8.3%)	0 (0%)	3 (10.3%)	3 (14.3%)	9 (8.2%)
Age					
Mean (SD)	21.5 (5.61)	21.1 (1.54)	21.8 (1.64)	24.4 (6.34)	22.0 (4.47)
Median [Min, Max]	20.0 [18.0, 48.0]	21.0 [19.0, 24.0]	21.0 [19.0, 25.0]	22.0 [21.0, 47.0]	21.0 [18.0, 48.0]
Religious					
Yes	7 (19.4%)	11 (45.8%)	8 (27.6%)	7 (33.3%)	33 (30.0%)
No	29 (80.6%)	13 (54.2%)	21 (72.4%)	14 (66.7%)	77 (70.0%)
Residence					
Rural	2 (5.6%)	1 (4.2%)	5 (17.2%)	2 (9.5%)	10 (9.1%)
Urban	34 (94.4%)	23 (95.8%)	24 (82.8%)	19 (90.5%)	100 (90.9%)
Knows someone with mental illness					
False	18 (50.0%)	12 (50.0%)	10 (34.5%)	14 (66.7%)	54 (49.1%)
True	18 (50.0%)	12 (50.0%)	19 (65.5%)	7 (33.3%)	56 (50.9%)
Relationship					
None	18 (50.0%)	12 (50.0%)	10 (34.5%)	14 (66.7%)	54 (49.1%)
Acquaintance	5 (13.9%)	1 (4.2%)	5 (17.2%)	3 (14.3%)	14 (12.7%)
Friend	2 (5.6%)	5 (20.8%)	8 (27.6%)	2 (9.5%)	17 (15.5%)
Family	11 (30.6%)	6 (25.0%)	6 (20.7%)	2 (9.5%)	25 (22.7%)
Frequency					
Not applicable	18 (50.0%)	12 (50.0%)	10 (34.5%)	14 (66.7%)	54 (49.1%)
Never	1 (2.8%)	1 (4.2%)	2 (6.9%)	1 (4.8%)	5 (4.5%)
Occasionally	6 (16.7%)	4 (16.7%)	10 (34.5%)	4 (19.0%)	24 (21.8%)
Monthly	4 (11.1%)	3 (12.5%)	1 (3.4%)	0 (0%)	8 (7.3%)
Weekly	3 (8.3%)	1 (4.2%)	5 (17.2%)	1 (4.8%)	10 (9.1%)
Daily	4 (11.1%)	3 (12.5%)	1 (3.4%)	1 (4.8%)	9 (8.2%)
History of mental health treatment					
False	21 (58.3%)	14 (58.3%)	21 (72.4%)	17 (81.0%)	73 (66.4%)
True	15 (41.7%)	10 (41.7%)	8 (27.6%)	4 (19.0%)	37 (33.6%)
Consider working in mental health field					
False	17 (47.2%)	13 (54.2%)	14 (48.3%)	8 (38.1%)	52 (47.3%)
True	19 (52.8%)	11 (45.8%)	15 (51.7%)	13 (61.9%)	58 (52.7%)

**Table 2:** Values of means and standard deviation for each subclass or dimension of stigmatising attitudes or behaviours of AQ-27 according to the participants’ socio-demographic characteristics.

	Anger		Avoidance		Blame		Coercion		Dangerousness		Fear		Help		Pity		Segregation	
	M	SD	M	SD	M	SD	M	SD	M	SD	M	SD	M	SD	M	SD	M	SD
Year of study																		
1st (N = 36)	10.47	5.15	14.64	6.15	8.64	5.05	16.58	4.05	11.75	5.6	10.44	5.75	21.69	3.98	17.78	3.99	10.83	6.06
2nd (N = 24)	11.83	7.28	15.92	6.42	13.54	6.87	18.46	5.36	13.88	6.74	15.08	7.55	21.33	3.61	20.96	4.48	12.83	6.93
3rd (N = 29)	8.79	5.83	12.97	7.27	10.66	5.36	17.24	4.89	11.9	5.14	10.38	6.06	22.48	4.04	18.14	4.21	10.59	6.12
4th (N = 21)	6	2.68	8.43	3.44	4.67	2.96	15.57	4.01	6.57	3.22	6.05	2.97	24.76	2.83	12.33	7.84	5	2.66
Sex																		
Female (N = 101)	9.33	5.72	13.18	6.59	9.38	5.92	16.76	4.7	11.24	5.79	10.59	6.46	22.49	3.95	17.63	5.59	10.12	6.25
Male (N = 9)	11.11	6.85	14.56	6.77	10.67	6.93	19.33	2.78	11.56	6.93	10.67	7.19	21.56	2.88	16.33	7.62	9.78	7.29
Religious																		
No (N = 77)	9.56	5.95	13.25	6.73	9.44	5.91	17.44	4.26	11.34	5.92	10.29	6.46	22.74	3.76	17.55	5.51	9.9	6.21
Yes (N = 33)	9.27	5.54	13.39	6.32	9.58	6.27	15.88	5.28	11.09	5.78	11.33	6.6	21.64	4.07	17.48	6.36	10.55	6.59
Residence																		
Rural (N = 10)	8.5	8.44	12	8.79	9.5	7.43	15.6	6.38	11.2	7.64	8.9	8.63	24.1	4.33	16.1	6.66	8.6	8.57
Urban (N = 100)	9.57	5.53	13.42	6.36	9.48	5.87	17.11	4.43	11.27	5.7	10.77	6.27	22.24	3.81	17.67	5.67	10.24	6.07
Relationship																		
None (N = 54)	10.26	6.71	13.31	7.44	9.91	7.15	18.02	4.88	11.5	6.99	10.98	7.2	22.56	4.42	17.19	6.82	10.65	7.5
Acquaintance (N = 14)	8.71	4.81	16.5	5.71	8.57	4.62	15.71	4.39	10.29	4.53	9.93	4.18	21.07	4.03	14.79	5.54	10.36	4.94
Friend (N = 17)	10.12	6.07	11.41	6.7	11.65	5.67	18.18	4.3	12.53	4.98	11.71	6.73	23.06	3.11	19.35	3.06	11.24	6.11
Family (N = 25)	7.76	3.41	12.72	4.23	7.6	3	14.6	3.37	10.44	4.2	9.4	5.86	22.4	2.87	18.56	4.05	7.96	3.42
History of mental health treatment																		
No (N = 73)	10.15	6	14.1	6.49	10.07	6.44	17.9	4.35	11.59	6.18	11.05	6.45	21.71	3.97	17.63	5.73	10.6	6.51
Yes (N = 37)	8.14	5.22	11.7	6.55	8.32	4.86	15.14	4.65	10.62	5.19	9.7	6.56	23.78	3.32	17.32	5.87	9.08	5.83
Consider working in mental health field																		
No (N = 52)	11.79	6.23	16	6.56	10.88	7.24	18.06	4.99	12.96	6.31	12.29	6.88	21.5	3.98	18.31	5.53	11.94	6.97
Yes (N = 58)	7.4	4.53	10.86	5.62	8.22	4.28	16	4.07	9.74	5	9.09	5.77	23.22	3.61	16.83	5.9	8.43	5.16
**Overall (N = 110)**	9.47	5.81	13.29	6.58	9.48	5.99	16.97	4.62	11.26	5.86	10.6	6.49	22.41	3.87	17.53	5.75	10.09	6.3

## Data Availability

The data presented in this study are available on request from the first author.

## Appendix A

**Table A1:** Values of minimum, maximum, mean and standard deviation, obtained for each of the AQ-27 items.

Question	Stereotype	Min	Max	Mean	SD
I would feel aggravated by Joseph.	Anger	1	9	3.74	2.07
How angry would you feel at Joseph?	Anger	1	9	4.25	2.19
How irritated would you feel by Joseph?	Anger	1	9	3.81	2.25
If I were an employer, I would interview Joseph for a job.	Avoidance	1	9	2.72	2.08
I would share a car pool with Joseph every day.	Avoidance	2	9	7.94	1.52
If I were a landlord, I probably would rent an apartment to Joseph.	Avoidance	1	9	3.95	2.37
I would think that it was Joseph’s own fault that he is in the present condition.	Blame	1	9	5.73	2.48
How controllable, do you think, is the cause of Joseph’s present condition?	Blame	2	9	7.54	1.75
How responsible, do you think, is Joseph for his present condition?	Blame	1	9	5.62	2.42
If I were in charge of Joseph’s treatment, I would require him to take his medication.	Coercion	1	9	2.18	2.18
How much do you agree that Joseph should be forced into treatment with his doctor even if he does not want to?	Coercion	1	9	4.65	2.36
If I were in charge of Joseph’s treatment, I would force him to live in a group home.	Coercion	1	8	3.02	2.14
I would feel unsafe around Joseph.	Dangerousness	1	9	3.86	2.28
How dangerous would you feel Joseph is?	Dangerousness	1	9	5.92	2.57
I would feel threatened by Joseph.	Dangerousness	1	9	3.45	2.27
Joseph would terrify me.	Fear	1	9	4.93	2.49
How scared of Joseph would you feel?	Fear	1	9	2.69	2.35
How frightened of Joseph would you feel?	Fear	1	9	3.15	2.09
I would be willing to talk to Joseph about his problems.	Help	1	9	3.35	2.27
How likely is it that you would help Joseph?	Help	4	9	7.75	1.29
How certain would you feel that you would help Joseph?	Help	3	9	7.13	1.55
I would feel pity for Joseph.	Pity	1	9	4.85	2.59
How much sympathy would you feel for Joseph?	Pity	1	8	2.65	2.29
How much concern would you feel for Joseph?	Pity	1	9	3.44	2.23
I think Joseph poses a risk to his neighbors unless he is hospitalised.	Segregation	1	9	3.12	2.32
I think it would be best for Joseph’s community if he were put away in a psychiatric hospital.	Segregation	1	9	6.05	2.38
How much do you think an asylum, where Joseph can be kept away from his neighbors, is the best place for him?	Segregation	1	9	7.06	2.01

### Appendix A.1. Anger

A Factorial ANOVA type 2 was performed to compare the main effects of Year of study, Sex, Religion, Residence, Relationship, History of mental health treatment and Consider working in the mental health field on the Box-Cox transformed score of Anger (λ = −0.1060) (Table A2). Analysis of the standardised residuals diagnostic plots, the Shapiro–Wilk normality test (W = 0.98, *p* = 0.092) and the Breusch–Pagan test (BP(11) = 13.21, *p* = 0.102), showed no clear violations of the normality and homoscedasticity assumptions of this model.

**Table A2:** Fixed-Effects ANOVA results using linkfun (Anger) as the criterion, where *p* values lower than the significance level of 0.05 are highlighted in bold, and LL and UL represent the lower-limit and upper-limit of the partial η^2^ confidence interval, respectively.

Predictor	Sum of Squares	*df*	Mean Square	*F*	*P*	Partial η^2^	Partial η^2^ 95% CI [LL, UL]
(Intercept)	21.40	1	21.40	126.66	0.000		
Year of study	3.18	3	1.06	6.27	0.001	0.16	[0.03, 0.27]
Sex	0.19	1	0.19	1.10	0.297	0.01	[0.00, 0.08]
Religion	0.04	1	0.04	0.27	0.608	0.00	[0.00, 0.06]
Residence	0.08	1	0.08	0.48	0.488	0.00	[0.00, 0.07]
Relationship	0.40	3	0.13	0.78	0.506	0.02	[0.00, 0.08]
History of mental health treatment	0.25	1	0.25	1.50	0.223	0.01	[0.00, 0.09]
Consider working in mental health field	2.43	1	2.43	14.39	0.000	0.13	[0.03, 0.25]
Error	16.56	98	0.17				

### Appendix A.2. Avoidance

A Factorial ANOVA type 2 was performed to compare the main effects of the Year of study, Sex, Religion, Residence, Relationship, Treatment and Psychiatry on the score of Avoidance (Table A3). Analysis of the standardised residuals diagnostic plots, the Shapiro–Wilk normality test (W = 0.90, *p* = 0.39) and the Breusch–Pagan test (BP(11) = 19.48, *p* = 0.05), showed no clear violations of the normality and homoscedasticity assumptions of this model.

**Table A3:** Fixed-Effects ANOVA results using Avoidance as the criterion, where *p* values lower than the significance level of 0.05 are highlighted in bold, and LL and UL represent the lower-limit and upper-limit of the partial η^2^ confidence interval, respectively.

Predictor	Sum of Squares	*df*	Mean Square	*F*	*P*	Partial η^2^	Partial η^2^ 95% CI [LL, UL]
(Intercept)	1420.71	1	1420.71	45.49	0.000		
Year of study	747.13	3	249.04	7.97	0.000	0.20	[0.06, 0.31]
Sex	52.12	1	52.12	1.67	0.199	0.02	[0.00, 0.10]
Religion	8.66	1	8.66	0.28	0.600	0.00	[0.00, 0.06]
Residence	0.43	1	0.43	0.01	0.907	0.00	[0.00, 0.02]
Relationship	185.49	3	61.83	1.98	0.122	0.06	[0.00, 0.14]
History of mental health treatment	31.95	1	31.95	1.02	0.314	0.01	[0.00, 0.08]
Consider working in mental health field	361.89	1	361.89	11.59	0.001	0.11	[0.02, 0.23]
Error	3060.54	98	31.23				

### Appendix A.3. Blame

A Factorial ANOVA type 2 was performed to compare the main effects of the Year of study, Sex, Religion, Residence, Relationship, History of mental health treatment and Consider working in the mental health field on the Box-Cox transformed score of Blame (λ = −0.2011) (Table A4). Analysis of the standardised residuals diagnostic plots, the Shapiro–Wilk normality test (W = 0.99, *p* = 0.445) and the Breusch–Pagan test (BP(11) = 6.09, *p* = 0.867), showed no clear violations of the normality and homoscedasticity assumptions of this model.

**Table A4:** Fixed-Effects ANOVA results using linkfun (Blame) as the criterion, where *p* values lower than the significance level of 0.05 are highlighted in bold, and LL and UL represent the lower-limit and upper-limit of the partial η^2^ confidence interval, respectively.

Predictor	Sum of Squares	*Df*	Mean Square	*F*	*P*	Partial η^2^	Partial η^2^ 95% CI [LL, UL]
(Intercept)	13.24	1	13.24	134.05	0.000		
Year of study	6.60	3	2.20	22.29	0.000	0.41	[0.24, 0.51]
Sex	0.17	1	0.17	1.74	0.191	0.02	[0.00, 0.10]
Religion	0.03	1	0.03	0.27	0.605	0.00	[0.00, 0.06]
Residence	0.00	1	0.00	0.00	0.994	0.00	[0.00, 1.00]
Relationship	0.48	3	0.16	1.61	0.193	0.05	[0.00, 0.13]
History of mental health treatment	0.17	1	0.17	1.70	0.195	0.02	[0.00, 0.10]
Consider working in mental health field	0.02	1	0.02	0.20	0.657	0.00	[0.00, 0.05]
Error	9.68	98	0.10				

### Appendix A.4. Coercion

Factorial ANOVA type 2 was performed to compare the main effects of the Year of study, Sex, Religion, Residence, Relationship, History of mental health treatment and Consider working in the mental health field on the score of Coercion (Table A5). Analysis of the standardised residuals diagnostic plots, the Shapiro–Wilk normality test (W = 0.99, *p* = 0.413) and the Breusch–Pagan test (BP(11) = 15.20, *p* = 0.174), showed no clear violations of the normality and homoscedasticity assumptions for this model.

**Table A5:** Fixed-Effects ANOVA results using Coercion as the criterion, where *p* values lower than the significance level of 0.05 are highlighted in bold, and LL and UL represent the lower-limit and upper-limit of the partial η^2^ confidence interval, respectively.

Predictor	Sum of Squares	*df*	Mean Square	*F*	*p*	Partial η^2^	Partial η^2^ 95% CI [LL, UL]
(Intercept)	1377.96	1	1377.96	81.65	0.000		
Year of study	160.97	3	53.66	3.18	0.027	0.09	[0.00, 0.19]
Sex	50.13	1	50.13	2.97	0.088	0.03	[0.00, 0.12]
Religion	60.78	1	60.78	3.60	0.061	0.04	[0.00, 0.13]
Residence	0.29	1	0.29	0.02	0.897	0.00	[0.00, 0.03]
Relationship	239.45	3	79.82	4.73	0.004	0.13	[0.02, 0.23]
History of mental health treatment	48.91	1	48.91	2.90	0.092	0.03	[0.00, 0.12]
Consider working in mental health field	21.58	1	21.58	1.28	0.261	0.01	[0.00, 0.09]
Error	1653.83	98	16.88				

### Appendix A.5. Dangerousness

A Factorial ANOVA type 2 was performed to compare the main effects of the Year of study, Sex, Religion, Residence, Relationship, History of mental health treatment and Consider working in the mental health field on the score of Box-Cox transformed score of Dangerousness (λ = 0.3453) (Table A6). Analysis of the standardised residuals diagnostic plots, the Shapiro–Wilk normality test (W = 0.99, *p* = 0.325) and the Breusch–Pagan test (BP(11) = 15.69, *p* = 0.153), showed no clear violations of the normality and homoscedasticity assumptions of these models.

**Table A6:** Fixed-Effects ANOVA results using linkfun (Dangerousness) as the criterion, where *p* values lower than the significance level of 0.05 are highlighted in bold, and LL and UL represent the lower-limit and upper-limit of the partial η^2^ confidence interval, respectively.

Predictor	Sum of Squares	*Df*	Mean Square	*F*	*p*	Partial η^2^	Partial η^2^ 95% CI [LL, UL]
(Intercept)	75.70	1	75.70	60.88	0.000		
Year of study	26.26	3	8.75	7.04	0.000	0.18	[0.05, 0.29]
Sex	0.21	1	0.21	0.17	0.682	0.00	[0.00, 0.05]
Religion	0.03	1	0.03	0.03	0.870	0.00	[0.00, 0.03]
Residence	0.04	1	0.04	0.03	0.866	0.00	[0.00, 0.03]
Relationship	2.29	3	0.76	0.62	0.607	0.02	[0.00, 0.07]
History of mental health treatment	0.23	1	0.23	0.18	0.668	0.00	[0.00, 0.05]
Consider working in mental health field	6.53	1	6.53	5.25	0.024	0.05	[0.00, 0.15]
Error	121.86	98	1.24				

### Appendix A.6. Fear

A Factorial ANOVA type 2 was performed to compare the main effects of the Year of study, Sex, Religion, Residence, Relationship, History of mental health treatment and Consider working in the mental health field on the Box-Cox transformed score of Fear (λ = 0.0169) (Table A7). Analysis of the standardised residuals diagnostic plots, the Shapiro–Wilk normality test (W = 0.99, *p* = 0.325) and the Breusch–Pagan test (BP(11) = 15.69, *p* = 0.153) showed no clear violations of the normality and homoscedasticity assumptions of these models.

**Table A7:** Fixed-Effects ANOVA results using linkfun (Fear) as the criterion, where *p* values lower than the significance level of 0.05 are highlighted in bold, and LL and UL represent the lower-limit and upper-limit of the partial η^2^ confidence interval, respectively.

Predictor	Sum of Squares	*Df*	Mean Square	*F*	*p*	Partial η^2^	Partial η^2^ 95% CI [LL, UL]
(Intercept)	23.65	1	23.65	69.49	0.000		
Year of study	8.11	3	2.70	7.94	0.000	0.20	[0.06, 0.31]
Sex	0.01	1	0.01	0.04	0.841	0.00	[0.00, 0.04]
Religion	0.35	1	0.35	1.04	0.311	0.01	[0.00, 0.08]
Residence	0.57	1	0.57	1.68	0.198	0.02	[0.00, 0.10]
Relationship	0.45	3	0.15	0.45	0.721	0.01	[0.00, 0.06]
History of mental health treatment	0.52	1	0.52	1.54	0.218	0.02	[0.00, 0.09]
Consider working in mental health field	1.02	1	1.02	2.99	0.087	0.03	[0.00, 0.12]
Error	33.36	98	0.34				

### Appendix A.7. Help

A Factorial ANOVA type 2 was performed to compare the main effects of the Year of study, Sex, Religion, Residence, Relationship, History of mental health treatment and Consider working in the mental health field on the Box-Cox transformed score of Help (λ = 2.9852) (Table A8). Analysis of the standardised residuals diagnostic plots, the Shapiro–Wilk normality test (W = 0.97, *p* = 0.014) and the Breusch–Pagan test (BP(11) = 14.73, *p* = 0.195), showed no clear violations of the normality and homoscedasticity assumptions for this model.

**Table A8:** Fixed-Effects ANOVA results using linkfun (Help) as the criterion, where *p* values lower than the significance level of 0.05 are highlighted in bold, and LL and UL represent the lower-limit and upper-limit of the partial η^2^ confidence interval, respectively.

Predictor	Sum of Squares	*Df*	Mean Square	*F*	*P*	Partial η^2^	Partial η^2^ 95% CI [LL, UL]
(Intercept)	45,425,647.47	1	45,425,647.47	20.06	0.000		
Year of study	42,269,057.61	3	14,089,685.87	6.22	0.001	0.16	[0.03, 0.27]
Sex	2,235,687.98	1	2,235,687.98	0.99	0.323	0.01	[0.00, 0.08]
Religion	13,598,139.32	1	13,598,139.32	6.01	0.016	0.06	[0.00, 0.16]
Residence	3,332,818.29	1	3,332,818.29	1.47	0.228	0.01	[0.00, 0.09]
Relationship	8,811,066.04	3	2,937,022.01	1.30	0.280	0.04	[0.00, 0.11]
History of mental health treatment	23,047,423.51	1	23,047,423.51	10.18	0.002	0.09	[0.01, 0.21]
Consider working in mental health field	2,653,178.32	1	2,653,178.32	1.17	0.282	0.01	[0.00, 0.08]
Error	221,909,016.33	98	2,264,377.72				

### Appendix A.8. Pity

A Factorial ANOVA type 2 was performed to compare the main effects of Year of study, Sex, Religion, Residence, Relationship, History of mental health treatment and Consider working in the mental health field on the score of Box-Cox transformed score of Pity (λ = 1.3199) (Table A9). Analysis of the standardised residuals diagnostic plots, the Shapiro–Wilk normality test (W = 0.98, *p* = 0.256) showed no clear violations of the normality and homoscedasticity assumptions of these models, and though the Breusch–Pagan test (BP(11) = 23.18, *p* = 0.016) indicated a deviation to homoscedasticity, this was not clear in the residuals plot.

**Table A9:** Fixed-Effects ANOVA results using linkfun (Pity) as the criterion, where *p* values lower than the significance level of 0.05 are highlighted in bold, and LL and UL represent the lower-limit and upper-limit of the partial η^2^ confidence interval, respectively.

Predictor	Sum of Squares	*df*	Mean Square	*F*	*P*	Partial η^2^	Partial η^2^ 95% CI [LL, UL]
(Intercept)	5501.05	1	5501.05	36.36	0.000		
Year of study	3635.74	3	1211.91	8.01	0.000	0.20	[0.06, 0.31]
Sex	0.55	1	0.55	0.00	0.952	0.00	[0.00, 1.00]
Religion	0.25	1	0.25	0.00	0.968	0.00	[0.00, 1.00]
Residence	10.26	1	10.26	0.07	0.795	0.00	[0.00, 0.04]
Relationship	643.75	3	214.58	1.42	0.242	0.04	[0.00, 0.12]
History of mental health treatment	174.15	1	174.15	1.15	0.286	0.01	[0.00, 0.08]
Consider working in mental health field	69.37	1	69.37	0.46	0.500	0.00	[0.00, 0.06]
Error	14,826.02	98	151.29				

### Appendix A.9. Segregation

A Factorial ANOVA type 2 was performed to compare the main effects of the Year of study, Sex, Religion, Residence, Relationship, History of mental health treatment and Consider working in the mental health field on the score of Box-Cox transformed score of Segregation (λ = 0.0987) (Table A10). Analysis of the standardised residuals diagnostic plots, the Shapiro–Wilk normality test (W = 0.98, *p* = 0.270) and the Breusch–Pagan test (BP(11) = 15.09, *p* = 0.178), showed no clear violations of the normality and homoscedasticity assumptions of these models.

**Table A10:** Fixed-Effects ANOVA results using linkfun (Segregation) as the criterion, where *p* values lower than the significance level of 0.05 are highlighted in bold, and LL and UL represent the lower-limit and upper-limit of the partial η^2^ confidence interval, respectively.

Predictor	Sum of Squares	*df*	Mean Square	*F*	*P*	Partial η^2^	Partial η^2^ 95% CI [LL, UL]
(Intercept)	26.86	1	26.86	50.83	0.000		
Year of study	14.05	3	4.68	8.87	0.000	0.21	[0.07, 0.33]
Sex	0.03	1	0.03	0.05	0.816	0.00	[0.00, 0.04]
Religion	0.04	1	0.04	0.07	0.786	0.00	[0.00, 0.04]
Residence	0.94	1	0.94	1.78	0.185	0.02	[0.00, 0.10]
Relationship	1.92	3	0.64	1.21	0.310	0.04	[0.00, 0.11]
History of mental health treatment	0.60	1	0.60	1.14	0.287	0.01	[0.00, 0.08]
Consider working in mental health field	1.59	1	1.59	3.01	0.086	0.03	[0.00, 0.12]
Error	51.78	98	0.53				
